# Mastectomy for Breast Cancer Under Regional Anesthesia Utilizing Intertransverse Process Block in a Patient Exhibiting Poor Pulmonary Function: A Case Report

**DOI:** 10.7759/cureus.78110

**Published:** 2025-01-28

**Authors:** Yuri Ishii, Takuya Yoshida, Hitoaki Sato, Norihiko Obata, Satoshi Mizobuchi

**Affiliations:** 1 Department of Anesthesiology, Konan Medical Center, Kobe, JPN; 2 Department of Anesthesiology, Kobe University Hospital, Kobe, JPN

**Keywords:** breast cancer, bronchiectasis, general anesthesia alternative, intertransverse process block, lung diseases, regional anesthesia

## Abstract

General anesthesia for patients with poor pulmonary function is a risk factor for perioperative pulmonary complications, and it should be avoided as much as possible, depending on the specifics of each case. Herein, anesthetic management for partial mastectomy in a patient with poor pulmonary function using intertransverse process block (ITPB) is reported. A woman in her 80s (height 149 cm, weight 45.9 kg) was scheduled to undergo a partial mastectomy for right breast cancer. She had bronchiectasis and interstitial pneumonia. ITPB was performed, and intravenous dexmedetomidine and lidocaine with epinephrine were administered locally. There was no worsening of dyspnea during surgery, and the patient was discharged on the seventh postoperative day without any complications.

## Introduction

The perioperative pulmonary complication rates range from 2% to 33%, with risk factors including older age, smoking, anemia, pulmonary or cardiac disease, and general anesthesia [1]. It is also recommended that general anesthesia be avoided whenever possible when the risk of perioperative pulmonary complications is high [1]. Recently, reports of intertransverse process block (ITPB) for breast surgery have received much attention [2,3]. However, there have been no reports of ITPB management of breast surgery in patients with poor pulmonary function. A case of partial mastectomy in a patient with poor pulmonary function performed under regional anesthesia by ITPB is reported. This article was previously presented at The 69th Kansai Branch Meeting of the Japan Society of Anesthesiologists on September 2, 2023.

## Case presentation

A woman in her 80s (height 149 cm, weight 45.9 kg) was diagnosed with right breast cancer four years earlier. She did not want treatment and was kept under observation. Since the right breast cancer was exposed on the body surface and began to bleed, and anemia progressed from Hb 13.8 to 9.2 g/dL, surgical treatment was planned. Her breast cancer was adenocarcinoma and classified as clinical stage IIIB, with no metastasis at that time. Her medical history included bronchiectasis and interstitial pneumonia. She was unable to lie flat in bed due to severe dyspnea and lived constantly in a semirecumbent or sitting position. Even simple conversations triggered wet coughs and shortness of breath, corresponding to Hugh-Jones classification V. She had no history of surgeries or allergies and was classified as American Society of Anesthesiologists (ASA) physical status III. Vital signs on admission were blood pressure 131/58 mmHg, heart rate 80/minute, respiratory rate 18/minute, and SpO_2_ 92% on room air. Arterial blood gas analysis showed partial pressure of oxygen 60.5 mmHg and partial pressure of carbon dioxide 46.1 mmHg on room air. Preoperative chest CT and X-ray images showed findings consistent with interstitial pneumonia and bronchiectasis, with no evidence of new pneumonia. The electrocardiogram showed sinus rhythm.

Considering the risk of pulmonary complications associated with general anesthesia, anesthetic management with a combination of regional anesthesia, local anesthesia, and sedation was planned. Due to the patient's poor respiratory condition and an estimated prognosis of less than one year, as well as the need to shorten the operation time, a discussion with the surgeon concluded that a right partial mastectomy for local control, rather than radical surgery, would be performed.

**Figure 1:**
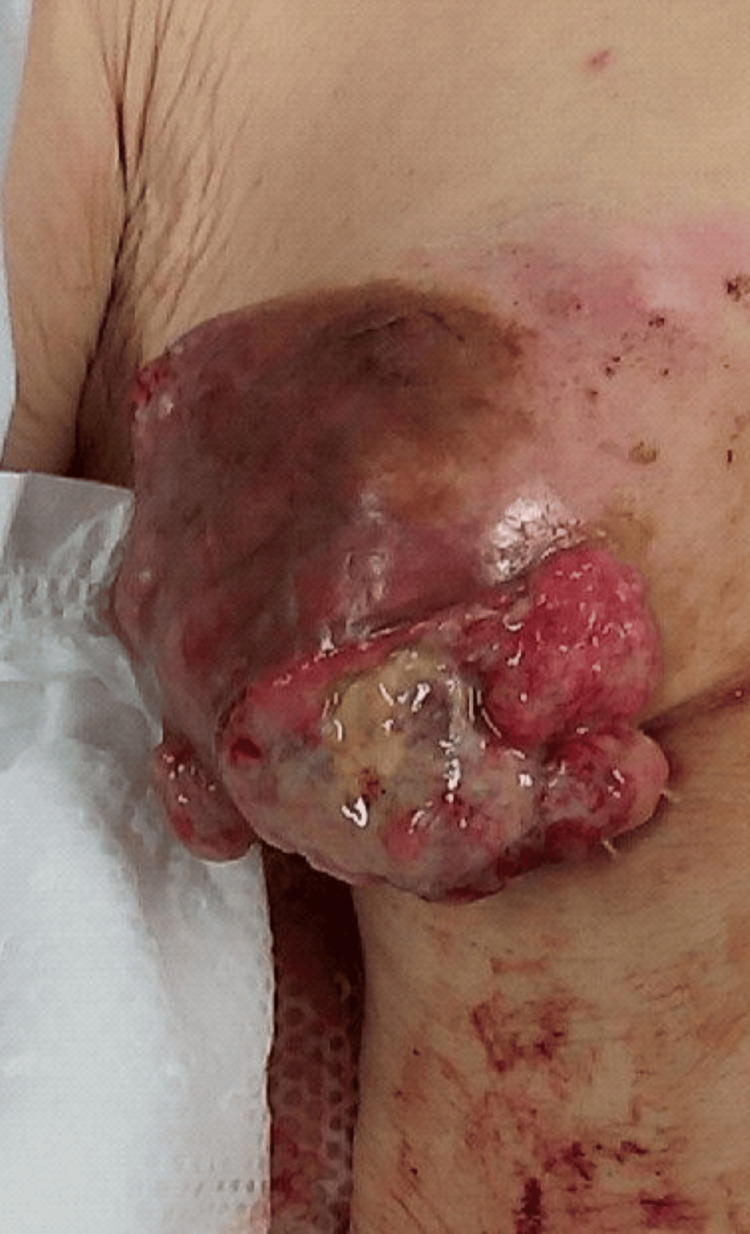
Ulcerated breast carcinoma being located in the anterior to right lateral thoracic region, which extends to the anterior axillary line

ITPB was selected as the regional anesthesia because the breast cancer lesion extended to the level of the anterior axillary line (Figure 1), to perform segmental anesthesia only on the affected side to reduce respiratory muscle depression, and to avoid the risk of pneumothorax. Written consent was obtained for anesthesia and surgery.

**Figure 2:**
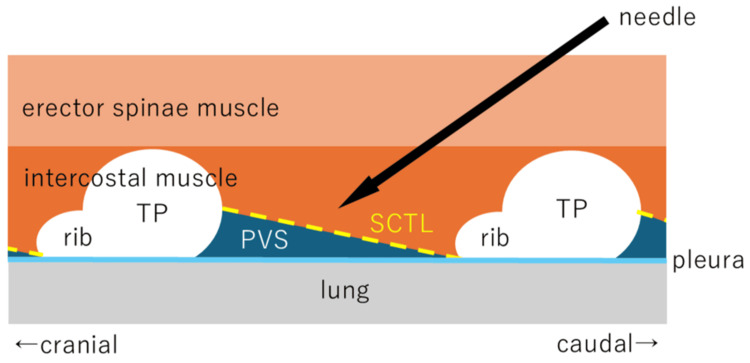
Schematic of ultrasound-guided ITPB With the transverse process and rib neck visible, identify the superior costotransverse ligament between the upper and lower transverse processes, and inject the local anesthetic into the dorsal aspect of the superior costotransverse ligament. The yellow broken line indicates SCTL TP: transverse process; SCTL: superior costotransverse ligament; PVS: paravertebral space; ITPB: intertransverse process block Image credit: This is an original image created by the authors Yuri Ishii, Takuya Yoshida, and Hitoaki Sato

Standard ASA monitors were attached and intravenous lines were secured in the operation room. Supplemental oxygen at 1 L/minute was administered via nasal cannula. After the patient was placed in the left lateral recumbent position, ITPB was done using a Sonosite Edge II ultrasound machine with a high-frequency linear probe HFL50x (15-6 MHz) (FUJIFILM Sonosite Inc., Bothell, WA). The echo probe was applied parallel to the spinal column, and the transverse costovertebral joint and the transverse processes of T2 to T6 and pleura were identified. A 23G, 60-mm Cattelan needle (Terumo Cattelan Needle®, Terumo, Tokyo, Japan) was then inserted in-plane from caudal to cranial, and the needle tip was placed at the midpoint between the transverse process and pleura (Figure 2). Then, 5 mL of 0.5% ropivacaine was injected at the T2/3 to T5/6 levels (four times, a total of 20 mL). Pleural displacement could be observed during drug injection.

After the block was performed, the patient was placed in a semirecumbent position. Sensory blockade of the incision site, previously identified in consultation with the surgeon, was confirmed by the cold test performed 15 minutes after the blocks, and the surgery was started. The patient was sedated with dexmedetomidine 0.7 µg/kg/hour during surgery. Since the patient complained of pain when surgical manipulation was performed on the head of the mammary gland and the pectoralis muscle, 0.25% and 0.125% lidocaine with epinephrine was administered locally as needed. The total lidocaine used was 50.7 mg. No other analgesics were required. Surgery time was 104 minutes, and anesthesia time was 143 minutes. During surgery, 1 L/minute oxygen was administered by nasal cannula, and SpO_2_ was maintained at 92%-99%. There was no worsening of dyspnea. Acetaminophen 15 mg/kg was administered at the end of surgery, and the patient was admitted to the intensive care unit. Four hours and 15 minutes after the end of the surgery, the patient complained of pain with a numerical rating scale score of 5 and was administered flurbiprofen axetil 50 mg. The patient was transferred to the general ward the day after surgery and discharged on the seventh postoperative day without any complications.

## Discussion

There have been reports that breast surgery can be managed under regional anesthesia alone [2-6], and the choice of anesthesia method should consider the surgical technique, tumor location, and complications associated with each type of anesthesia. This is the first report of breast surgery in a patient with poor pulmonary function that was managed under regional anesthesia with ITPB.

Epidural anesthesia and paravertebral block (PVB) have long been widely used as regional anesthesia for breast surgery. Singh et al. reported 50 breast surgeries that were managed with epidural anesthesia alone and noted that, although postoperative recovery was good and medical costs were low, there was a risk of dural puncture and postdural puncture headache [4]. Simpson et al. reported that PVB alone was effective in managing 28 breast surgery cases with ischemic heart disease and pulmonary disease [5].

In recent years, there have been case reports of mastectomy management using ITPB rather than epidural anesthesia or PVB [2,3]. The reason for avoiding general anesthesia was patient preference in both reports. Costache reported a case managed with ITPB and sedation with propofol, and Marrone et al. reported a case managed with ITPB and erector spinae plane block (ESPB) and sedation with dexmedetomidine and ketamine. A mid-point transverse process to pleura block has also been reported, and it is now called ITPB.

Because the present patient had extremely poor pulmonary function and was elderly, partial mastectomy rather than radical surgery was planned to avoid general anesthesia and anemia due to bleeding. In selecting the regional anesthesia technique, it was considered that it should provide the necessary analgesic area and not cause a decrease in pulmonary function or pneumothorax, and the necessary punctures could be done. First, upper thoracic epidural anesthesia has been reported to cause a 20%-30% decrease in lung volume and volume per second due to paralysis of bilateral intercostal muscles [7], and it was excluded because of its potentially significant impact on the present patient. Second, pneumothorax has been reported to occur in three per 1,000 breast surgeries in which ultrasound-guided PVB was performed [8]; although the probability of pneumothorax occurring is low, PVB was excluded because the risk nevertheless remains. Third, because the lesion site extended from the anterior to the lateral thoracic region, pectoral nerve block II, transversus thoracic muscle plane block, and serratus anterior plane block, which are approached from the anterior thoracic region, were excluded. Because ITPB is effective on only one side, has a lower risk of causing pneumothorax than PVB, and can be approached from the back, it was selected in the present case.

ITPB is a nerve block technique reported by Costache et al. [9]. It is similar to PVB, where the anesthetic is delivered to the paravertebral space, which acts on the intercostal nerve roots. However, the risk of pneumothorax is considered lower than with PVB because the needle tip is located at the midpoint between the posterior transverse process and the pleura.

Concerning the method of drug administration in ITPB, there is debate as to whether single or multiple doses are better; Nielsen et al. performed ITPB in 12 healthy men and compared a single 21 mL dose of 0.75% ropivacaine with three doses of 7 mL each, and reported no difference in the analgesic range [10]. In a cadaveric validation, Costache et al. injected 5 mL of dye each and reported that it often reached the paravertebral space at the adjacent level [9]. On the other hand, Ohgoshi et al. injected 20 mL of dye and reported that penetration into the paravertebral space was observed only in the intercostal area where the dye was injected [11]. According to previous reports of mastectomy managed with ITPB, both reports used a single dose, but Costache used a single dose because of the narrow extent of the resection [2], and Marrone et al. added ESPB because of the large extent [3]. In the present case, it was necessary to ensure analgesia in the Th2/3 to 5/6 range to avoid general anesthesia, and multiple doses were selected.

The sensory nerves of the breast include the intercostal nerves, the supraclavicular nerve innervating the upper chest, and the pectoral nerve innervating the pectoralis major muscle. Because ITPB does not block these nerves arising from the cervical or brachial plexus, additional local anesthesia is required when surgical manipulations involve the upper chest or thoracic muscles. In the present case, this was explained to the surgeon in advance, and he was asked to administer additional local anesthetics as needed, which facilitated the completion of the surgery.

Furthermore, when surgical manipulation involves the axillary region, an ITPB at the T2-6 level, as performed in this case, may not provide sufficient analgesia. Therefore, it would be necessary to perform ITPB at higher levels or add another analgesic method. Munasinghe et al. reported a case in which an intercostobrachial nerve block combined with a supraclavicular brachial plexus block was used for axillary clearance in a patient with asthma [6]. This suggests that adding an intercostobrachial nerve block could be a valuable option for achieving effective analgesia during axillary clearance.

## Conclusions

We experienced anesthetic management for partial mastectomy in a patient with poor pulmonary function. ITPB reduced the risk of pneumothorax and provided adequate analgesia, and she was discharged without exacerbation of dyspnea or pulmonary complications. ITPB may be an option for anesthetic management of breast surgery for patients with poor pulmonary function.
